# Successful application of extracorporeal membrane oxygenation treatment in the management of severe respiratory failure caused by primary pulmonary lymphoma: A case report

**DOI:** 10.1097/MD.0000000000028717

**Published:** 2022-01-28

**Authors:** Chao Yu, Lunbing Xv, Xiaochun Peng, Min Shao

**Affiliations:** Department of Critical Care Medicine, The First Affiliated Hospital of Anhui Medical University, PR China.

**Keywords:** case report, extracorporeal membrane oxygenation, primary pulmonary lymphoma, respiratory failure

## Abstract

**Rationale:**

Primary pulmonary lymphoma (PPL) is a rare disease, and rapid progression of pulmonary exudation leads to severe respiratory failure. Here, we present the case of a critically ill patient with PPL complicated by refractory hypoxemic respiratory failure. The patient was ultimately cured with a successful combination of extracorporeal membrane oxygenation (ECMO) and chemotherapy.

**Patient concerns:**

A 36-year-old woman was hospitalized because of a 2-month history of cough with fever and shortness of breath. Computed tomography revealed multiple pulmonary nodules, consolidation, and solid pulmonary opacities. Complications of pneumothorax occurred after computed tomography-guided core needle biopsy, and respiratory failure progressively developed (PaO_2_/FiO_2_ 65 mm Hg).

**Diagnosis:**

Primary pulmonary lymphoma, respiratory failure, stress cardiomyopathy, cardiogenic shock.

**Interventions:**

The patient was treated with veno-venous ECMO and chemotherapy.

**Outcomes:**

The patient was successfully weaned off ECMO after chemotherapy and transferred out of the intensive care unit on day 9. After regular chemotherapy, no obvious lesions were observed in either lung tissue.

**Conclusion:**

ECMO can be selected as an important salvage treatment for patients with severe cardiopulmonary dysfunction caused by PPL and other malignant tumors that may be cured or transferred to a stable stage.

## 1 Introduction

Extracorporeal membrane oxygenation (ECMO) is a type of advanced life support technology in which venous blood is withdrawn from a patient and sent through a membrane lung where oxygen is supplied and carbon dioxide is removed. Subsequently, blood was returned to the patient.^[1]^ Treatment of refractory hypoxemic respiratory failure is the primary indication for ECMO. According to the Extracorporeal Life Support Organization (ELSO) guidelines, there are no absolute contraindications for patients with malignancy undergoing ECMO according to the ELSO guidelines.^[2]^ However, in clinical practice, ECMO is rarely used for patients with malignancies. ECMO is frequently associated with complications and injuries, specifically bleeding, infections, and thrombocytopenia. Primary pulmonary lymphoma (PPL) is a rare disease that accounts for 0.5% of all primary lung tumors and 1% of all lymphomas.^[3]^ Clinical and radiological features of PPL are nonspecific. In some patients with PPL, the rapid progression of pulmonary exudation leads to severe respiratory failure. Here, we present the case of a critically ill patient with PPL complicated by refractory hypoxemic respiratory failure. The patient was ultimately cured by a successful combination of ECMO and chemotherapy, which is the first reported case. The patient provided informed consent for the publication of this case.

## 2 Case presentation

**Figure 1:**
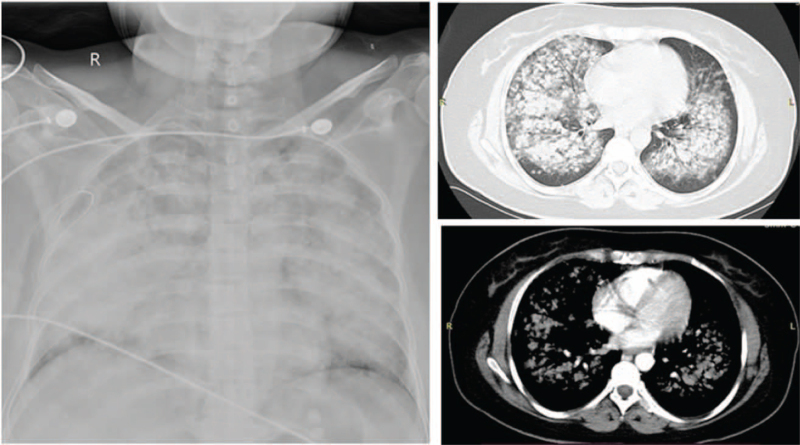
A chest radiography and computed tomography (CT) indicated multiple pulmonary nodules with air bronchograms, consolidation and solid pulmonary opacities.

**Figure 2:**
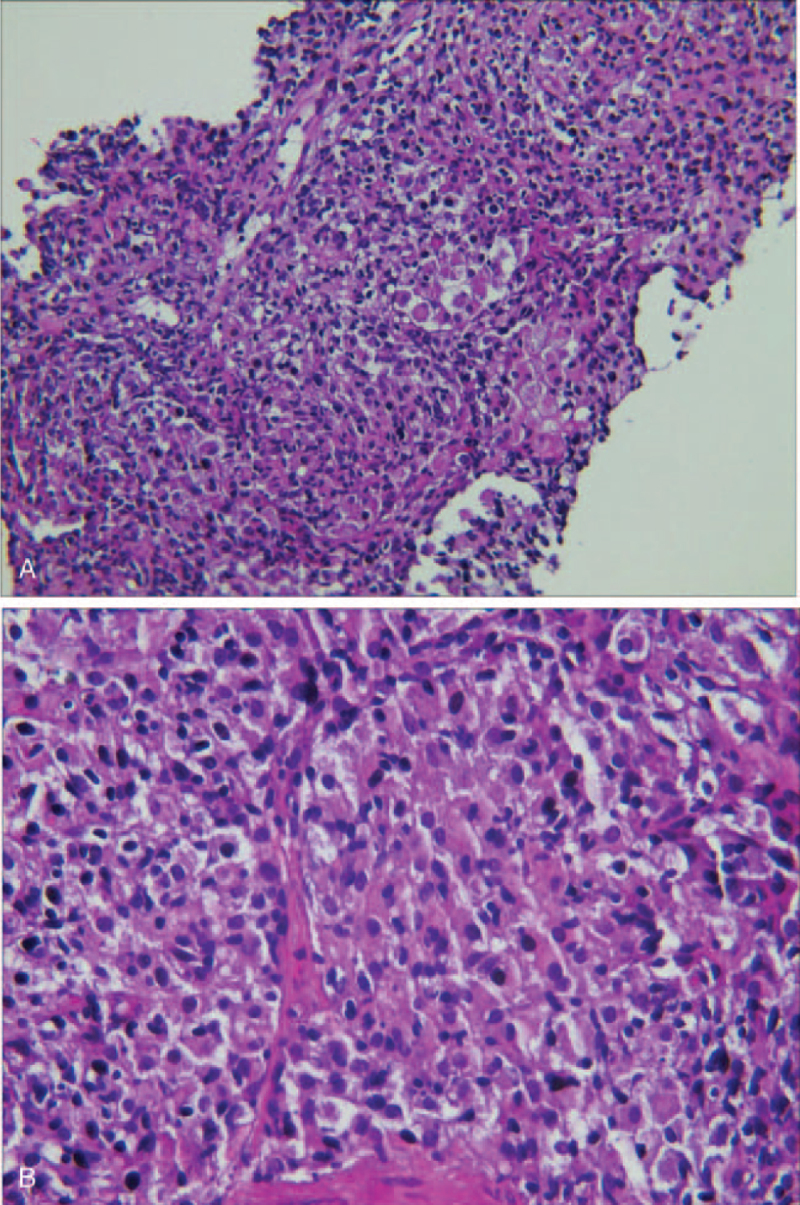
There was a large amount of histiocytic reaction in the alveolar lumen and moderate lymphocytic infiltration in the interstitium (H&E A: 100× B:400×.

**Figure 3:**
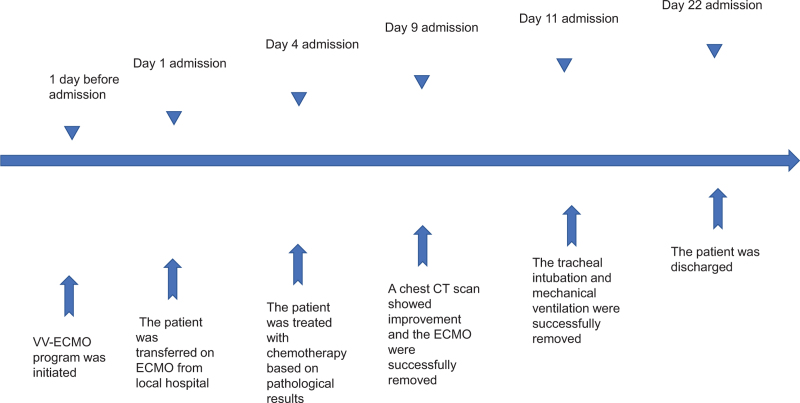
Treatment and efficacy of the patient.

**Figure 4:**
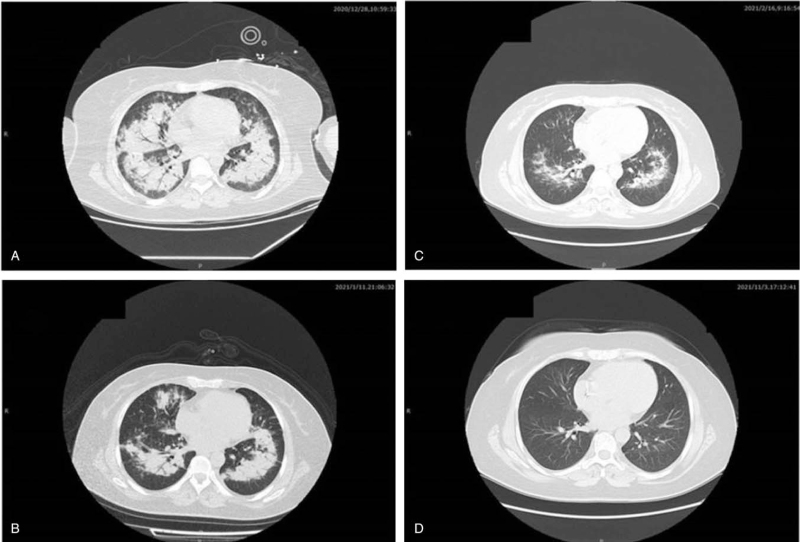
Imaging. (A) The 9th day after admission: multiple pulmonary nodules, consolidation. (B) The 22th day after admission: there were few consolidation in both lungs. (C) The 57th day after admission: there were few exudative lesions and no consolidation in both lungs. (D) The 316th day after admission: no obvious lesions were found in either lung.

A 36-year-old woman was hospitalized with a 2-month history of a cough and fever. Five days previously, the patient's symptoms had deteriorated. She manifested shortness of breath and was admitted to the Pneumology Department of a local hospital (200 km away from our hospital) in December 2020. The patient's husband denied any medical history. Chest radiography and computed tomography (CT) revealed multiple pulmonary nodules, consolidation, and solid pulmonary opacities (Fig. 1). The patient was treated with levofloxacin and cefoperazone-sulbactam; however, the patient failed to improve. On the 7th day after admission to the local hospital, a CT guided core needle biopsy was performed. Unfortunately, postoperative complications such as pneumothorax have occurred. Closed thoracic drainage was immediately performed, but the peripheral capillary oxygen saturation (SpO_2_) was as low as 80% to 90% (high-flow nasal cannula FiO_2_: 80%). The patient was transferred to the ICU for endotracheal intubation and ventilation (PCV mode was set to PC 18 cmH_2_O, PEEP 12 cmH_2_O, and FiO_2_ 100%). However, respiratory failure progressively developed (PaO_2_/FiO_2_ 65 mm Hg, Murray score 15). The patient developed severe hypoxia, stress cardiomyopathy (left ventricular ejection fraction: 30%), and cardiogenic shock (Video S1, Supplemental Digital Content) and required a high dose of norepinephrine (1.2 μg/kg∗min) to maintain blood pressure. Our hospital's ECMO team was rushed to the local hospital. The patient was placed on femoral veno-venous ECMO (VV-ECMO). After the guidewire was inserted percutaneously, the patient was systemically heparinized. Fifty units per kilogram heparin intravenous injection, and 2 to 10 units of heparin per kilogram per hour intravenous pumping to maintain activated partial thromboplastin time 1.4 to 1.5 times normal. The ECMO speed was 3500 rpm, with a flow rate of 4 to 4.5 L/min, BP of 111/62 mm Hg, and SpO_2_ of 90%. After her vital signs stabilized, she was transferred to the ICU of our hospital for further treatment. After transfer to our ICU, the patient's cardiac function gradually recovered after the stress factors were eliminated (Video 2, Supplemental Digital Content) and the norepinephrine dose gradually decreased. On the third day of admission to our ICU, the results of the lung biopsy in the local hospital returned. The pathological results showed moderate lymphocyte infiltration into the alveolar interstitium (Fig. 2). The immunohistochemical results were as follows: CD3 (−), CD43 (+), CD4 (+), CD8 (−), CD20 (−), CD56 (−), TIA-1 (+), EBER-ISH (−), CK7 (alveolar epithelial cells+), and CD68 (histocytes+). No abnormalities were found in the bone marrow biopsy. Based on the morphological and immunophenotypic findings, as well as the clinical data, primary pulmonary lymphoma was diagnosed by hematology consultation. The patient was then treated with chemotherapy (CHOP), which included cyclophosphamide, epirubicin, vincristine, and prednisolone, on the fourth day of admission to our ICU (fifth day after ECMO was performed). During treatment, lung oxygenation steadily improved. Following the suspension of ECMO airflow (flow rate of 3 L/min) on day 9 after ICU admission, SPO_2_ was maintained at 95% to 98% with the assistance of a ventilator (FiO_2_, 40%), and the oxygenation index was more than 250 mm Hg. The follow-up chest CT scan (9th day after admission) showed partial improvement (Fig. 3), and the ECMO cannulas were removed. On the 11th day after ICU admission, tracheal intubation and mechanical ventilation were successfully removed. She was sent to the Department of Hematology for additional treatment 2 days later and was discharged on the 22nd day after admission (Fig. 3). After regular chemotherapy, the chest CT showed improvement (Fig. 4).

## 3 Discussion

Although the lung is frequently involved in systemic lymphoma, primary pulmonary lymphoma accounts for less than 1% of all extranodal lymphomas. B-cell lymphomas are the most common type of PLL, and T-cell lymphomas are very rare in the lungs.^[4]^ Patients with PLL may manifest nonspecific clinical symptoms such as cough, fever, chest pain, dyspnea, and hemoptysis. Chest CT is the first choice for imaging diagnosis. Radiological features also manifest non-specifically, including pulmonary nodules, consolidation, and solid pulmonary opacities.^[3]^ Primary pulmonary lymphomas can develop rapidly and cause severe respiratory failure.

Since its inception, ECMO has been a popular rescue treatment for patients with severe hypoxemia. The initial indication was acute reversible heart or lung disease in patients who were likely to die. Malignant tumors were considered a contraindication for ECMO. However, the indications for ECMO are continuously changing. At present, there are no specific ECMO guidelines for patients with tumors, and there is no consensus on the indications for ECMO for adult critically ill patients. However, it is generally accepted that VV-ECMO should be considered as a treatment for reversible respiratory failure with a high risk of mortality.^[5]^ The PaO_2_/FiO_2_ ratio was 65 mm Hg and the Murray score was 15 in this case report, and it was a clear indication for patients undergoing VV-ECMO.

ECMO has recently been used to treat acute cardiopulmonary failure in patients with malignant tumors.^[6–8]^ Cancer frequently experience serious complications from malignancy or treatment. Kenneth et al examined the ELSO Registry database for adults with malignancies from 1992 to 2008. Seventy two adults met the inclusion criteria, and 23 (32%) patients with malignancy required ECMO primarily for cardiopulmonary support and survived until hospital discharge.^[9]^ Wohlfarth et al published a retrospective study in 2014 of 14 adult patients with hematologic malignancies who were all on ECMO for acute respiratory failure. After an average of 8.5 days on ECMO, 50% of the patients survived ICU stay. This study found that shorter time intervals between ICU admission and the start of ECMO and shorter ECMO duration had a major influence on higher survival rates.^[6]^ The patient in this case report survived severe respiratory failure mainly due to the early use of ECMO, and no serious infection occurred before or during ICU admission.

Prior to this, the patient underwent ECMO. The patient had severe respiratory failure and cardiogenic shock caused by stress cardiomyopathy. Respiratory failure was more serious than shock. Therefore, VV-ECMO was selected at the local hospital, and the subsequent treatment proved that the choice was correct. Echocardiography showed an improvement in systolic function with adequate oxygen delivery. In addition, it is worth mentioning that pneumothorax was only an aggravation of respiratory failure in this patient. The patient persistently suffered from severe hypoxia even when the local hospital successfully provided closed thoracic drainage at the time of the first pneumothorax diagnosis.

In summary, we reported a case of primary pulmonary lymphoma-related severe respiratory failure that was successfully treated with ECMO. Patients with PPL may benefit from the active use of ECMO to sustain oxygen transportation and have sufficient time to receive chemotherapy treatment, especially if the tumor is rapidly progressing. ECMO can be selected as an important salvage treatment for patients with severe cardiopulmonary dysfunction caused by PPL and other malignant tumors that may be cured or transferred to a stable stage.

## Acknowledgments

We are grateful to all participants of this study. We thank Dr. Huaiping Yuan for technical assistance.

## Author contributions

**Conceptualization:** Chao Yu, Min Shao.

**Data curation:** Chao Yu, Xiaochun Peng, Lunbing Xv.

**Methodology:** Chao Yu, Min Shao.

**Writing – original draft:** Chao Yu.

**Writing – review & editing:** Chao Yu, Min Shao.

## Supplementary Material

Supplemental Digital Content

## Supplementary Material

Supplemental Digital Content
